# Comparison of In Vitro Killing Activity of Rezafungin, Anidulafungin, Caspofungin, and Micafungin against Four *Candida auris* Clades in RPMI-1640 in the Absence and Presence of Human Serum

**DOI:** 10.3390/microorganisms9040863

**Published:** 2021-04-16

**Authors:** Renátó Kovács, Zoltán Tóth, Jeffrey B. Locke, Lajos Forgács, Gábor Kardos, Fruzsina Nagy, Andrew M. Borman, László Majoros

**Affiliations:** 1 Department of Medical Microbiology, Faculty of Medicine, University of Debrecen, 4032 Debrecen, Hungary; kovacs.renato@med.unideb.hu (R.K.); toth.zoltan@med.unideb.hu (Z.T.); forgacs.lajos.89@gmail.com (L.F.); kg@med.unideb.hu (G.K.); nagyfruzsina0429@gmail.com (F.N.); 2 Doctoral School of Pharmaceutical Sciences, University of Debrecen, 4032 Debrecen, Hungary; 3 Cidara Therapeutics, Inc., 6310 Nancy Ridge Dr., Suite 101, San Diego, CA 92121, USA; jlocke@cidara.com; 4 UK National Mycology Reference Laboratory, Public Health England, Science Quarter, Southmead Hospital, Bristol BS10 5NB, UK; andy.borman@nbt.nhs.uk; 5 Medical Research Council Centre for Medical Mycology (MRC CMM), University of Exeter, Exeter EX4 4QD, UK

**Keywords:** *Candida auris*, rezafungin, echinocandin, killing rate, time kill, serum

## Abstract

*Candida auris* is an emerging and frequently multidrug-resistant pathogen against which the echinocandins are the preferred therapeutic option. We compared killing activities of anidulafungin, caspofungin, micafungin, and rezafungin against 13 isolates representing four *C. auris* clades (South Asian *n* = 3; East Asian *n* = 3; South African *n* = 3; South American *n* = 4, of which two were of environmental origin). Minimum inhibitory concentration MICs and killing kinetics in RPMI-1640 and RPMI-1640 plus 50% serum (50% serum) were determined. The four echinocandins were never fungicidal and induced large aggregates in RPMI-1640 and, less markedly, in 50% serum. Colony forming unit CFU decreases were found more consistently in 50% serum than in RPMI-1640. Isolates from the East Asian clade were killed at ≥1–≥ 4 mg/L with all echinocandins regardless of media. Anidulafungin and micafungin produced killing at peak drug serum concentration (8 mg/L) against environmental but not clinical isolates from the South American and the South African clades. Micafungin at ≥8 mg/L but not anidulafungin produced CFU decreases against the South Asian clade as well. In 50% serum, rezafungin at ≥1–≥ 8 mg/L produced killing against all four clades. The next generation echinocandin, rezafungin, showed the same or better activity at clinically attainable trough concentration regardless of media, compared with anidulafungin, caspofungin, and micafungin against all four tested *C. auris* clades.

## 1. Introduction

*Candida auris* is an emerging fungal pathogen causing asymptomatic yet ubiquitous colonization and life-threatening invasive infections among critically ill patients in intensive care units [1]. Based on whole-genome sequence data of global clinical isolates, five phylogenetically distinct clades (South Asian, East Asian, South African, South American, and Iranian) were discovered to have emerged simultaneously on different continents [2,3]. *C. auris* lineages differ in their microscopic self-aggregating ability, growth in the presence of actidione, and ability to produce pseudomycelium on Dalmau cultures [4,5]; furthermore, significant clade-specific differences in virulence between South Asian, East Asian, South African, and South American lineages were detected using invertebrate and neutropenic murine models [4,6,7]. This pathogen is almost always multidrug resistant, leaving echinocandins (anidulafungin, caspofungin, and micafungin) as the current first-line therapeutic option for the treatment of *C auris* infections [1,8,9].

The echinocandins act as noncompetitive inhibitors of the β-1,3-d-glucan synthase, which is responsible for the biosynthesis of β-1,3-d-glucan, an essential structural component of fungal cell walls. In vitro, echinocandins show fungicidal or fungistatic activity against *Candida* species, including azole-resistant *C. krusei* or *C. glabrata* isolates [10]. The efficacy of echinocandins correlates with the ratios of area under the concentration curve per minimum inhibitory concentration (AUC/MIC) or serum peak drug concentration per minimum inhibitory concentration (C_max_/MIC). All echinocandins are highly protein bound (≥97.5%), which decreases the free thus active drug concentration, which may affect standard susceptibility testing in vitro. Echinocandin resistance is rare and is associated with mutations in FKS genes, which encode the catalytic subunit of β-1,3-d-glucan synthase [10].

Rezafungin is a next-generation echinocandin with distinctive pharmacokinetics, including prolonged half-life (>130 h) and once-weekly intravenous administration [11,12,13]. An initial dose of 400 mg rezafungin produces 22.7 mg/L C_max_ and 1160 mg·h/L AUC values in the first week, pharmacokinetic parameters, which are 2.95- to 3.24-fold and 1.4- to 1.9-fold higher, respectively, compared to C_max_ and AUC values produced by standard daily doses of anidulafungin, caspofungin, and micafungin [10,12]. In vitro, rezafungin activity is comparable to that of the three approved echinocandins against common and uncommon *Candida* species, including *C. auris* [14,15]. However, data on killing kinetics against different *C. auris* clades are absent. Therefore, we compared the killing activities of anidulafungin, caspofungin, micafungin, and rezafungin against the four prevalent *C. auris* clades (South Asian, East Asian, South African, and South American). Since rezafungin, similar to other echinocandins, is highly protein bound with 0.2–3% free drug, killing activity was determined in standard RPMI-1640 medium and in RPMI-1640 containing 50% human serum (50% serum) [16].

## 2. Materials and Methods

### 2.1. Isolates

All isolates, representing four *C. auris* clades (South Asian *n* = 3, East Asian *n* = 3, South African *n* = 3, South American *n* = 4), derived from our previous study (Table 1) [6]. Strains were stored at −70 °C. Two days before the in vivo experiments, isolates were subcultured using Sabouraud agar and screened on CHROMagar *Candida* (Becton Dickinson) to ensure the purity of *Candida* isolates.

### 2.2. Antifungal Susceptibility Testing

Rezafungin pure powder was provided by Cidara Therapeutics (San Diego, CA, USA). Caspofungin, micafungin, and anidulafungin were obtained from Molcan Corporation (Richmond Hill, ON, Canada). Minimum inhibitory concentration (MIC) values were determined simultaneously using the standard Clinical Laboratory Standars Institute (CLSI) broth macrodilution method in RPMI-1640 and in RPMI-1640 supplemented with 50% serum (human serum from a human male, type AB, not heat inactivated, Sigma, Budapest, Hungary) [16,17,18,19]. Antifungals were dissolved in 100% DMSO and diluted further in RPMI-1640 to final concentrations between 0.015–8 mg/L. MICs were read visually after 24 h using the partial inhibition criterion. For categorization, tentative MIC breakpoints as suggested by the Centers for Disease Control and Prevention were used: ≤4 mg/L for anidulafungin, ≤2 mg/L for caspofungin, and ≤4 mg/L for micafungin [9].

### 2.3. Time-Kill Studies

The killing activity was determined in RPMI-1640 with and without 50% human serum at concentrations 1–32 mg/L and at 0.25–32 mg/L, respectively, in a final volume of 10 mL [19]. These echinocandin ranges were based on previous pharmacokinetic data with standard doses of currently approved echinocandins among intensive care unit patients [20,21,22] and Phase 1 data for rezafungin [12]. The starting inocula were 2.5–7 × 10^5^ CFU/mL. Aliquots of 100 mL were removed at 0, 4, 8, 12, and 24 h, serially diluted ten-fold, plated (4 × 30 mL) onto a single Sabouraud dextrose agar, and incubated at 35 °C for 48 h. All experiments were performed in both media twice [19].

Killing kinetics at the tested concentrations were analyzed in both media (RPMI and RPMI-1640 plus 50% serum), as described previously. Positive killing rate (*k*) values indicate killing, and negative *k* values indicate growth. Since echinocandins never produced a fungicidal effect against *C. auris* isolates, only the mean times to achieve a 50% reduction of the starting inoculum (T_50_ = 0.30103/*k*) were calculated from the *k* values for each isolate and concentration in both media [19].

One-way ANOVA with Tukey’s post-testing was used to determine significant differences in killing kinetics among isolates and concentrations in either RPMI-1640 or 50% serum. T-test (with Welch’s correction, where appropriate) was used for the same echinocandin concentrations in RPMI-1640 and 50% serum to determine significant differences in killing kinetics in the different media [19].

### 2.4. Phase-Contrast Microscopy

Echinocandin-induced morphological alterations were examined at 1 and 16 mg/L with two isolates from each clade (isolates 12, 27, 15, 12,372, 204, 2, 13,108, and I-172) with all echinocandins after 24 h of incubation at 37 °C in both media, with a Zeiss Axioskop 2 mot microscope coupled with a Zeiss Axiocam HRc camera using the phase-contrast technique. Image acquisition was performed, using Zeiss Axiovision 4.8.2 software. The total volume examined was 10 µL.

## 3. Results

### 3.1. MIC Values of the Echinocandins against C. auris

In RPMI-1640, MICs of anidulafungin, caspofungin, and micafungin were not higher than the suggested tentative breakpoints for *C. auris* regardless of clades (Table 1) [9]. Rezafungin showed comparable MICs with the approved echinocandins. In 50% serum, MICs were higher with all four drugs, with the lowest range (0.25–1 mg/L) observed for rezafungin, which may be related to relatively lower protein binding [10,16]. MIC increases in serum were the lowest (1- to 8-fold) and highest (8- to 64-fold) with caspofungin and anidulafungin, respectively.

### 3.2. Time-Kill Studies

**Figure 1:**
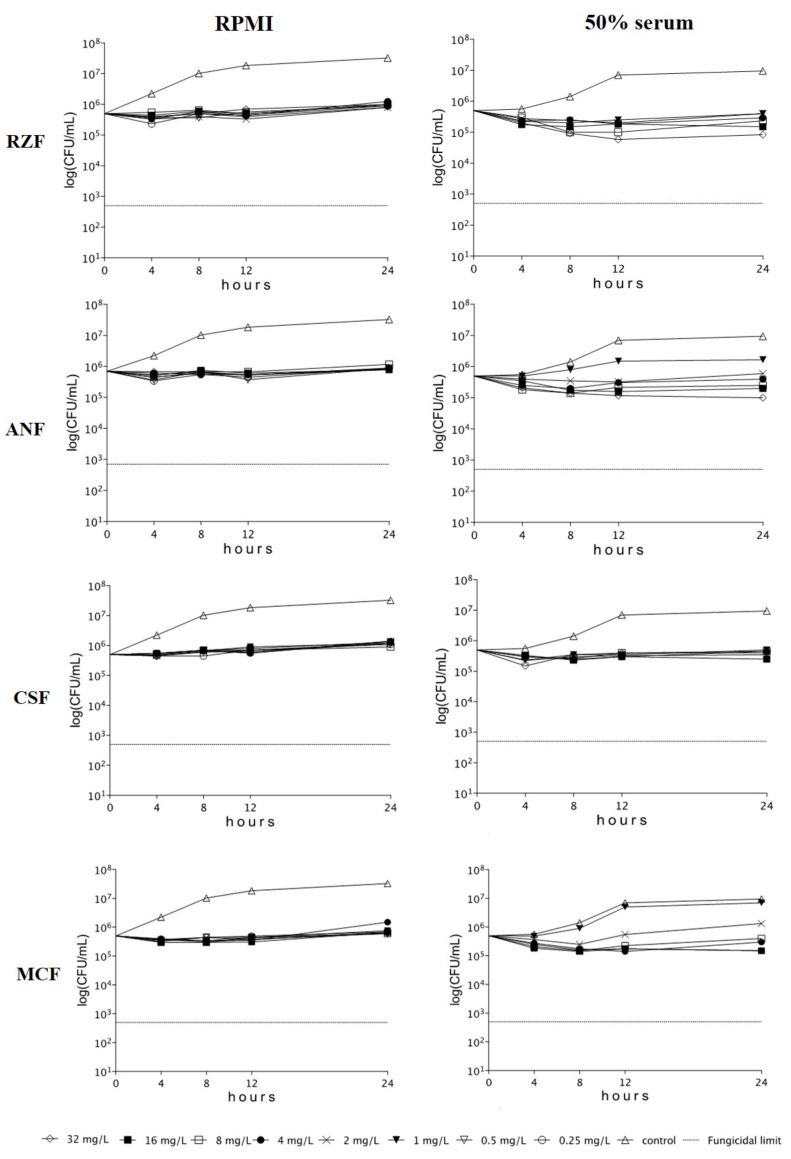
Time-kill plots of rezafungin (RZF), anidulafungin (ANF), caspofungin (CSF), and micafungin (MCF) in RPMI-1640 (RPMI) and RPMI-1640 plus 50% human serum (50% serum) against *Candida auris* isolate I-172, belonging to the South American clade.

All four echinocandins, regardless of clade and medium (RPMI-1640+/−50% serum), were fungistatic (less than 99.9% reduction in viable cell count, compared to the starting inoculum) against *C. auris* isolates. Inhibition of isolates was frequently found only in the first 8–12 h with prominent regrowth after 24 h. Time-kill plots of isolate I-172 from the South American clade are observed in both media in Figure 1.

### 3.3. South Asian Clade

**Figure 2:**
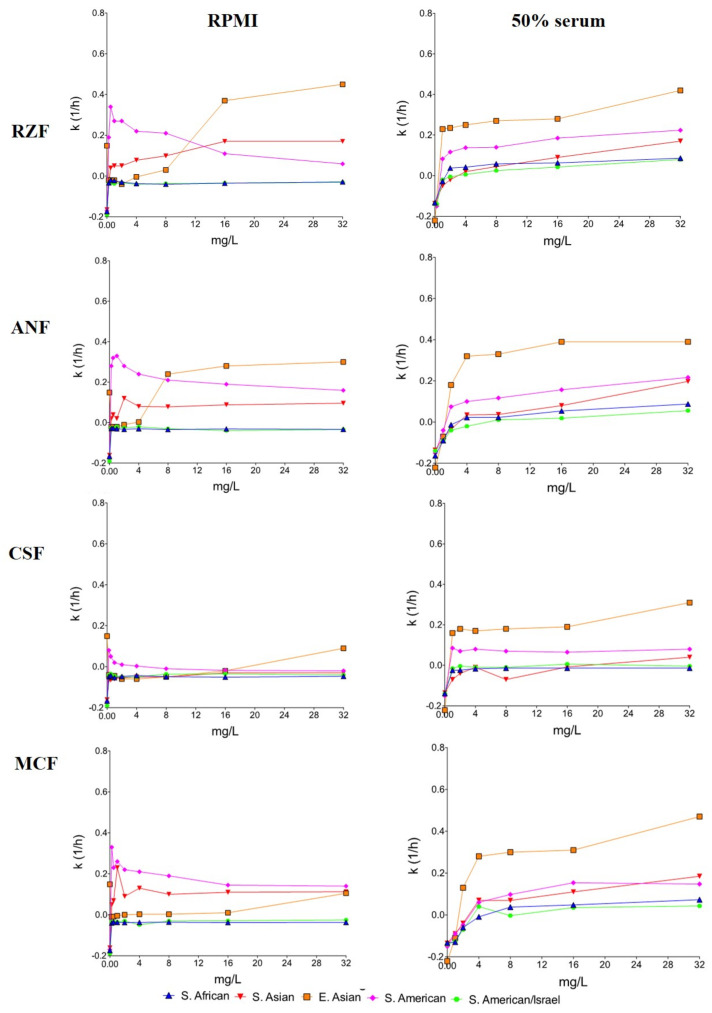
Mean killing rate values of rezafungin (RZF), anidulafungin (ANF), caspofungin (CSF), and micafungin (MCF) in RPMI-1640 (RPMI) and RPMI-1640 plus 50% human serum (50% serum) against 4 *Candida auris* clades. Data of the type strain (NCPF 13029 = CBS 10913) were not included in the East Asian clade. Positive and negative *k* values indicate the decrease and increase, respectively, in viable cell numbers. Error bars were omitted for better visualization of the graphics.

In RPMI-1640, the mean killing rates were concentration dependent at 0.25–32 mg/L for rezafungin, anidulafungin, and micafungin. However, only rezafungin at 4–32 mg/L and anidulafungin at 16–32 mg/L produced uniformly positive *k* values against the three isolates (Table 2 and Figure 2). Rezafungin *k* values, in the case of isolate 27 at 0.25–2 mg/L, were higher (0.22–0.27 1/h) than at 4–32 mg/L (0.10–0.14 1/h) (mini-paradoxical effect).

In 50% serum, rezafungin, anidulafungin, and micafungin at 4–32 mg/L (≥2–4× MIC) produced concentration-dependent killing activity without regrowth (*p* > 0.05 comparing these three echinocandins). In 50% serum, the mean anidulafungin *k* value at 32 mg/L (*k* = 0.20 1/h) was higher than in RPMI-1640 (*k* = 0.10 1/h) (*p* < 0.05). Caspofungin *k* values were positive against all three isolates only at 32 mg/L (mean *k* value was 0.04 1/h.) (Table 2 and Figure 2).

### 3.4. East Asian Clade

In RPMI-1640, rezafungin, anidulafungin, and caspofungin at 0.25–32 mg/L showed concentration-dependent killing activity against the *C. auris* type strain, producing colony forming unit (CFU) decreases close to the fungicidal limit at 32 mg/L (2.6–2.9 log decrease) (data not shown). Micafungin showed greater killing activity at 0.25–2 mg/L (*k* value ranges were 0.21–0.25 1/h), compared to 4–32 mg/L (*k* values were 0.11–0.14 1/h) (mini-paradoxical growth). In 50% serum, killing activity of rezafungin and caspofungin was concentration dependent at 1–32 mg/L. Micafungin and anidulafungin generated positive *k* values at ≥4 mg/L and ≥2 mg/L, respectively.

For the remaining two isolates in RPMI-1640, the mean *k* values at lower concentrations (0.25–4 mg/L) were less consistently positive than in 50% serum; in RPMI-1640, positive *k* values were noticed against both isolates only with rezafungin and anidulafungin at 8–32 mg/L. In contrast, in 50% serum, rezafungin and caspofungin at 1–16 mg/L, anidulafungin at 2–32 mg/L, and micafungin at 4–32 mg/L produced concentration-independent killing activity against the two isolates (Table 2 and Figure 2).

### 3.5. South African Clade

In RPMI-1640, South African isolates were inhibited but never killed by either of the four echinocandins, even at 256–1024× MIC. The highest CFU decreases (−0.4 log) was noticed with 1 mg/L anidulafungin against isolate 185 after 12 h (Figure 2).

In 50% serum, the killing activity of echinocandins increased against all isolates. However, against all isolates, only rezafungin produced positive *k* values (mean *k* values at 8–32 mg/L were 0.07–0.09 1/h) (Table 2 and Figure 2).

### 3.6. South American Clade

The isolates of the different origin in this clade behaved differently, i.e., bloodstream isolates from Israel showed a markedly different response to echinocandins from the behavior of Colombian environmental isolates. Israeli isolates were similar to the South African clade; in RPMI-1640, *k* values were always negative (no killing) at all tested concentrations (Figure 2). In 50% serum, rezafungin at 8–32 mg/L and anidulafungin and micafungin at 32 mg/L showed positive *k* values against both isolates (mean *k* values were 0.03–0.08, 0.06, and 0.04 1/h, respectively) (Table 2 and Figure 2). Caspofungin always produced negative *k* values with the two isolates at all tested concentrations.

Against the hospital environmental isolates from Colombia (isolates 13,108 and 16,565), all four echinocandins showed significantly greater killing at lower, than at higher, concentrations (mini-paradoxical effect) in RPMI-1640 (Table 2 and Figure 2). In 50% serum, rezafungin, anidulafungin, and micafungin showed concentration-dependent killing, while caspofungin showed concentration-independent killing against the two isolates. In serum, rezafungin produced positive *k* values even at ≥1 mg/L against both isolates (Figure 2).

### 3.7. Phase-Contrast Microscopy

**Figure 3:**
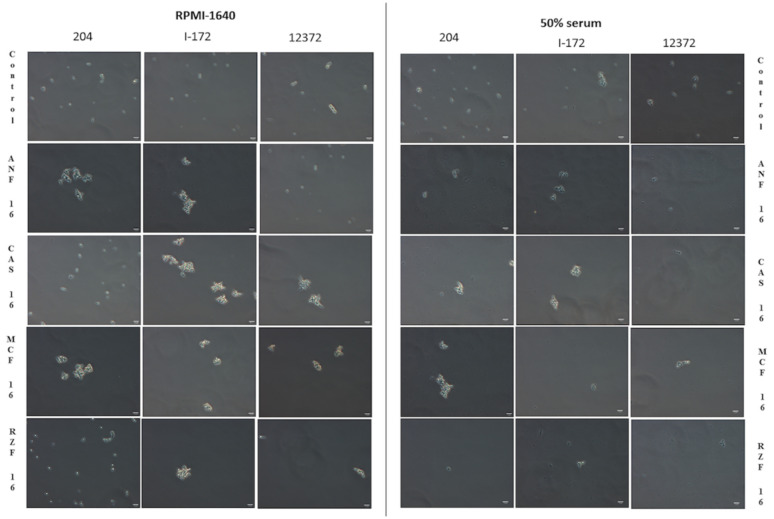
Phase-contrast microscopy images of untreated *C. auris* cells and cells treated with 16 mg/L rezafungin (RZF), 16 mg/L anidulafungin (ANF), 16 mg/L caspofungin (CSF), and 16 mg/L micafungin (MCF) in RPMI-1640 and RPMI-1640 plus 50% human serum (50% serum) against isolates 204 (South African clade), I-172 (South American clade) and 12,372 (East Asian clade). The micrograph was taken after 24 h. In RPMI-1640, single and budding cells or short chains with small aggregates containing up to 3–6 cells were observed in controls. In 50% serum, the appearance of the untreated isolates was similar, but small aggregates (up to 5–10 µm in diameter) were more frequently observed. In RPMI-1640, all four echinocandins induced large aggregates in the case of isolate I-172, and ANF and MCF in the case of isolate 204, while CSF and MCF in the case of isolate 12372. In 50% serum, large aggregates could be observed only in cases of isolate 204 with CSF and MCF, and isolate I-172 with CSF. Abnormal cell morphology and cell debris were typical after 24 h. Bar: 10 µm.

Echinocandin-treated *C. auris* cells in RPMI-1640 frequently showed large (20–30 µm in diameter) aggregates with up to 100 cells, regardless of the clade. Neither the number nor the size of the aggregates was dependent on the echinocandin or its concentration (i.e., any echinocandin at 1 or 16 mg/L was able to induce large aggregates). In 50% serum, the number and size of aggregates decreased significantly at both concentrations. Abnormal cell morphology and cell debris were typical after 24 h at 16 mg/L regardless of the echinocandin or clade (Figure 3).

## 4. Discussion

This study demonstrated that in RPMI-1640, after 24 h, the four echinocandins did not produce killing at 32 mg/L against the South African clade and isolates from Israel (South American clade). These isolates responded similarly to echinocandin exposure despite their significantly different virulence in a neutropenic murine model (South African isolates showed the least, while isolates from Israel the highest virulence) [6]. The two environmental isolates from the South American clade (Colombian isolates) were killed at ≥0.25 mg/L, but mini-paradoxical growth was observed with all four echinocandins (Table 2). Against the two East Asian clinical isolates and South Asian clade, only rezafungin and anidulafungin (at ≥4-≥8 mg/L and ≥8-≥16 mg/L, respectively) produced CFU decreases after 24 h. However, all four echinocandins showed good killing activity against the *C. auris* type strain (NCPF 13029; CBS 10913).

Although MIC values increased in the presence of 50% human serum, the killing activity of echinocandins increased against all clades at ≥2× MICs. In addition, in 50% serum, the occurrence of echinocandin-induced aggregates decreased significantly regardless of the clade (Figure 3). The killing activity of caspofungin was negligible, except against the East Asian clade and the two isolates from the South American clade. Anidulafungin and micafungin proved to be effective at peak concentration (~8 mg/L) [20,22] against isolates from Colombia (South American clade) but not against isolates from Israel (South American clade) or the South African clade. The next-generation rezafungin showed the same or greater activity, at clinically attainable trough concentration (4 mg/L) regardless of media, than did anidulafungin and micafungin against the South Asian, East Asian, and South American (Colombian isolates) clades [12,20,22]. Moreover, a single 400-mg dose of rezafungin produces ≥8 mg/L serum levels even after 48–72 h, which is capable of early enhanced killing and sustained therapeutic effect to maintain elimination of the fungus, including isolates from Israel (South American clade) and the South African clade, against which other echinocandins were less active [12].

Data on in vitro killing activity of echinocandins against *C. auris* are scant. Dudiuk et al. [23] determined the killing activity of caspofungin and anidulafungin in RPMI-1640 against 9 *C. auris* bloodstream isolates from Colombia. They reported slightly poorer killing, compared to our Colombian isolates (South American clade) (average *k* value ranges for caspofungin and anidulafungin were between −0.006 to +0.07 1/h and 0.005 to 0.07 1/h, respectively). The lack of similar studies comparing rezafungin to the three approved echinocandins against different *C. auris* clades precludes comparative discussion of our results.

An important finding of our study is that, regardless of media, significant differences exist between in vitro killing by the currently approved echinocandins and by rezafungin against the four prevalent *C. auris* clades. MIC values do not reflect the weak inhibition without killing effect against some *C. auris* clades. One possible explanation is that echinocandin exposure, regardless of medium, clade, or isolate, induces large aggregates of *C. auris,* as demonstrated both in vitro and in vivo [4,5,6]. We have previously demonstrated that *C. auris* isolates from certain clades (particularly the South African clade) grow as large aggregates that cannot be physically disrupted by sonication, vortex mixing, or with detergents, and have proposed that this phenotype is due to failure to release daughter cells and complete abscission after cell division [4]. In addition, we have shown that this phenotype can also be reversibly induced in isolates of “nonaggregative” clades by exposure to low concentrations of those antifungal agents that affect cell wall and cell membrane integrity [5]. This phenotype is clearly due to defects in cytokinesis, which are constitutive in certain clades and drug inducible in others, rather than a physical clumping of individual cells, due to, for example, changes in cell surface properties [4,5]. This phenomenon has also been seen in vivo [4,6]. The aggregate formation may be a general survival strategy of *C. auris*, and yeast cells in the center of the aggregates could be protected from the antifungals and from immune effectors. Aggregate formation in vitro is necessary but not sufficient for *C. auris* cells to survive echinocandin exposure since a significant proportion of cells were killed in both media, even in the case of the more resistant isolates from Israel.

Another explanation for the weak fungistatic effect of echinocandins is that decreased amount of β-glucan induces a variety of stress adaptation pathways resulting in increased cell wall chitin [10,24,25,26]. Increased chitin content helps to stabilize the fungal cell wall, therefore mitigating the impact of echinocandins. Although this adaptive response does not lead to mutations in the *FKS* genes, the early adaptation to echinocandin exposure enhances survival of the drug effect and may allow the development of stable resistance mechanisms over time for surviving cells, possibly leading to clinical resistance in the long run [24,25,26]. The mini-paradoxical effect in RPMI-1640, which disappeared in the case of some isolates in the presence of serum, may represent such adaptation. The high protein binding decreases the free (i.e., active) echinocandin concentration as modeled by the presence of 50% serum, which is mirrored in elevated MICs [10]. Lower free drug levels induce less compensatory chitin synthesis; thus, higher *k* values at higher concentrations with serum may be explained by the enhancement of the killing activities of echinocandins due to lower cell wall chitin, compared to RPMI-1640 (Figure 2) [10,26]. Although chitin content was not measured in our study, other authors found elevated chitin content in *C. auris*, compared to other species more susceptible to echinocandins [27]. Furthermore, fast increase of chitin content in response to caspofugin was reported with caspofungin-resistant but not caspofungin-susceptible *C. auris* isolates [28].

The in vivo efficacy of the four echinocandins was not determined in animal models using these isolates, which may be regarded as a limitation. However, other authors found that a human equivalent dose of micafungin (5 mg/kg daily) given early (2 h post-infection) was effective in decreasing the fungal kidney burdens in a neutropenic murine model against echinocandin-susceptible *C. auris* isolates from different countries [29]. Similar results were reported with a humanized dose of rezafungin (single 20 mg/kg) against three *C. auris* isolates in a neutropenic murine model [30]. A combination of echinocandins with new triazoles may be a promising approach to improve the therapeutic efficacy further against *C. auris* [31,32].

An important implication of our findings, i.e., the currently used standard echinocandin regimens do not reliably produce serum concentrations high enough to eradicate *C. auris* from the bloodstream, may partially explain therapeutic failures among critically ill patients. This may also contribute to the continuous candidemia and unacceptably high mortality among SARS-CoV2–positive patients with *C. auris* infections in spite of treatment with standard doses of echinocandins [9,29,33]. The very high C_max_ and AUC values produced by once-weekly rezafungin in the blood and tissues and its excellent safety profile even at supratherapeutic doses (>400 mg weekly) in healthy subjects, together with promising findings in animal models [12,34], raise hopes that rezafungin may prove superior to the earlier echinocandins in treatment of life-threatening *C. auris* infections.

## 5. Conclusions

In vitro killing activities of echinocandins against *C. auris* were clade-, isolate-, and medium dependent. All four echinocandins induced large aggregates in RPMI-1640 and 50% serum, and killing in 50% serum was more consistently positive than in RPMI-1640. The next-generation echinocandin, rezafungin, showed the same or greater activity at clinically attainable trough concentration regardless of media, compared with anidulafungin, caspofungin, and micafungin against the South Asian, East Asian, South American, and South African clades.

## Figures and Tables

**Table 1:** Minimum inhibitory concentration (MIC) values of rezafungin (RZF), anidulafungin (ANF), caspofungin (CSF), and micafungin (MCF) in RPMI-1640−/+ 50% human serum against *Candida auris* isolates and type strain. MICs were determined three times using the Clinical Laboratory Standars Institute broth macrodilution method.

Isolates Number	Clade	Body Site	MIC Values in RPMI/RPMI + 50% Serum (mg/L)			
			RZF	ANF	CSF	MCF
12	South Asian	unknown	0.03–0.06/0.5	0.12/2	1/0.5–1	0.12–0.25/2
20 (NCPF 8985)	South Asian	Wound	0.25/0.5	0.25/1	1/2	0.25/2
27 (NCPF 89891)	South Asian	Pleural fluid	0.12/1	0.12/2	0.5/2	0.25/2
Type strain (NCPF 13029 = CBS 10913)	East Asian	External ear	0.06/0.5	0.03/0.5–1	0.25/0.5	0.12/2–4
15 (NCPF 8984)	East Asian	External ear	0.03/0.25–0.5	0.03–0.06/2	0.5–1/1	0.12/2
12372 (CBS 12372)	East Asian	Blood	0.06/0.5	0.03/0.5–1	0.12/0.5	0.03/2
204	South African	Tracheostomy	0.06/0.25-0.5	0.03/2	0.25/0.5–1	0.12–0.25/2
2 (NCPF 8977)	South African	Cerebrospinal fluid	0.12/1	0.03/2	0.5/1	0.25/2–4
185	South African	Blood	0.12/1	0.25/2	0.25/1	0.25/4
I-24	South American (Israel)	Blood	0.25/0.5	0.06/4	0.25/1	0.12/4
I-172	South American (Israel)	Blood	0.25/0.5	0.06/2	0.25/1	0.12/2
13108 (CDC B-13108)	South American (Colombia)	Hospital environment	0.12/0.5	0.06/2	0.25/1	0.12/4
16565 (CDC B-16565)	South American (Colombia)	Hospital environment	0.12/0.25	0.015/0.5	0.25/0.5	0.06/2

**Table 2:** Average time (hours) to reach 50% growth reduction (T_50_ = 0.30103/*k*) from the starting inocula at different rezafungin (RZF), anidulafungin (ANF), caspofungin (CSF), and micafungin (MCF) concentrations in RPMI-1640 −/+ 50% human serum against 4 *Candida auris* clades. Data of the type strain (NCPF 13029 = CBS 10913) were not included in the data of the East Asian clade. Data in bold indicate that *k* values were positive (indicating killing) for all isolates within a clade, all other data (nonbold) indicate that one or two isolates never reached 50% growth reduction; the datum represents the average of those isolates in which the 50% growth reduction was reached. NA: 50% growth inhibition not achieved; ND: not determined.

50% Serum	Clade	Drug	Time (Hours)							
			32 mg/L	16 mg/L	8 mg/L	4 mg/L	2 mg/L	1 mg/L	0.5 mg/L	0.25 mg/L
**(−)**	South Asian (3 isolates)	RZF	**1.77**	**1.77**	**3**	**3.75**	1.36	1.09	1.23	1.19
		ANF	**3.1**	**3.4**	3.4	2.14	2.14	3.51	1.22	1.82
		MCF	1.54	1.62	1.56	1.27	1.58	0.75	2	2.06
		CSF	NA	NA	NA	NA	NA	NA	NA	NA
	East Asian (2 isolates)	RZF	**0.67**	**0.81**	**10**	23	NA	15.5	15.3	16.4
		ANF	**1**	**1.07**	**1.25**	30.1	24.6	19.3	21.6	13.8
		MCF	1.3	6.95	1.56	13.6	18	31.6	69.7	NA
		CSF	1.38	NA	NA	NA	NA	NA	NA	NA
	South African (3 isolates)	RZF	NA	NA	NA	NA	NA	NA	NA	NA
		ANF	NA	NA	NA	NA	NA	NA	NA	NA
		MCF	NA	NA	NA	NA	NA	NA	NA	NA
		CSF	NA	NA	NA	NA	NA	NA	NA	NA
	South American (2 isolates from Israel)	RZF	NA	NA	NA	NA	NA	NA	NA	NA
		ANF	NA	NA	NA	NA	NA	NA	NA	NA
		MCF	NA	NA	NA	NA	NA	NA	NA	NA
		CSF	NA	NA	NA	NA	NA	NA	NA	NA
	South American (2 isolates from Colombia)	RZF	**5**	**2.7**	**1.43**	**1.36**	**1.11**	**1.11**	**0.88**	**1.58**
		ANF	**1.58**	**1.42**	**1.15**	**1.08**	**1.5**	**0.91**	**0.71**	**1.25**
		MCF	**2.14**	**2.07**	**1.58**	**1.43**	**1.36**	**1.15**	**1.3**	**0.91**
		CSF	NA	NA	NA	100.3	29.4	**15**	**6**	**3.75**
**(+)**	South Asian (3 isolates)	RZF	**1.77**	**3.34**	**6.69**	**15.1**	75.3	NA	ND	ND
		ANF	**1.5**	**3.75**	**7.5**	**7.5**	NA	NA	ND	ND
		MCF	**1.58**	**2.73**	**4.29**	**4.29**	NA	NA	ND	ND
		CSF	**3.75**	75	NA	NA	NA	NA	ND	ND
	East Asian (2 isolates)	RZF	**0.71**	**1.08**	**1.11**	**1.2**	**1.25**	**1.3**	ND	ND
		ANF	**0.77**	**0.77**	**0.91**	**0.94**	**1.67**	NA	ND	ND
		MCF	**0.64**	**0.97**	**1**	**1.07**	0.97	NA	ND	ND
		CSF	**0.97**	**1.58**	**1.97**	**1.77**	**1.97**	**1.88**	ND	ND
	South African (3 isolates)	RZF	**3.42**	**3.81**	**4.3**	1.85	1.88	100	ND	ND
		ANF	2.04	3.01	2.08	2.48	6.53	NA	ND	ND
		MCF	2.73	3.75	4.29	7.3	NA	NA	ND	ND
		CSF	17.2	43.8	NA	NA	21.1	NA	ND	ND
	South American (2 isolates from Israel)	RZF	**3.76**	**7.07**	**10.03**	12.57	38.2	39.2	ND	ND
		ANF	**5.38**	7.17	7.61	80.1	NA	NA	ND	ND
		MCF	**7**	4.27	24.6	9.56	NA	NA	ND	ND
		CSF	NA	17.3	NA	NA	67.3	NA	ND	ND
	South American (2 isolates from Colombia)	RZF	**1.37**	**1.63**	**2.14**	**2.17**	**2.57**	**3.64**	ND	ND
		ANF	**1.39**	**1.92**	**2.57**	1.41	1.89	15.1	ND	ND
		MCF	**2.04**	**1.96**	**3.08**	1.76	45.5	NA	ND	ND
		CSF	1.81	2.15	1.95	1.78	2.01	1.74	ND	ND

## Data Availability

Data are available from the corresponding author upon reasonable request.
